# Guidelines and recommendations for preparing policy briefs from research into policy-making in health sciences: a scoping review

**DOI:** 10.1186/s13643-026-03090-4

**Published:** 2026-02-09

**Authors:** Marie Derstroff, Luisa Schmidt, Tim Mathes, Eni Shehu, Charlotte Kugler, Martin Bujard, Helena Ludwig-Walz, Dawid Pieper

**Affiliations:** 1 Faculty of Health Sciences Brandenburg, Brandenburg Medical School (Theodor Fontane, Institute for Health Services and Health System Research, Rüdersdorf, Germany; 2 Center for Health Services Research,, Brandenburg Medical School (Theodor Fontane), Rüdersdorf, Germany; 3 Institute for Quality and Efficacy in Health Care, Department of Health Economics, Cologne, Germany; 4 Department of Medical Statistics, University Medical Center Göttingen, Göttingen, Germany; 5 Federal Institute for Population Research (BiB), Wiesbaden, Germany; 6 Institute for Medical Psychology, Medical Faculty, Heidelberg University, Heidelberg, Germany

**Keywords:** Health policy, Policy-making, Knowledge translation, Public health, Policy brief

## Abstract

**Objective:**

This scoping review aims to provide an overview of documents describing the preparation of policy briefs from academic health science researchers for policy-making.

**Introduction:**

Not considering research evidence sufficiently may engender inefficient resource allocation and an inadequate response to public issues. Policy briefs are aimed at bridging this gap and assisting policy-makers in making evidence-informed decisions, yet they lack standardization.

**Methods:**

A scoping review following JBI methodology was conducted. We aimed at summarizing recommendations and guidelines for policy briefs from health science academia, focusing on formal criteria and contextual considerations. We included documents describing the preparation of policy briefs from academia to policy-making, as well as those that are usable for various addressees. Documents needed to be published in German, English, French, or Spanish and available in full text. Searches were conducted in PubMed, Embase, Web of Science, LIVIVO (SOMED), and additional sources up to December 01, 2025, resulting in 67 included records out of 1395 scientific publications and 81 grey literature sources.

**Results:**

The structure of policy briefs varied, with layout and language being the only consistent elements. Guidelines exhibited diversity in length, target groups, references, and timing, with discernible tendencies. Contextual considerations also varied across articles, indicating inconsistency in definitions and frequencies.

**Conclusion:**

Variability in policy brief design guidance poses challenges for both researchers and end-users, potentially hindering effective evidence communication. Enhanced efforts of co-creation, shared minimum standards, and evaluation may mitigate these challenges. Still, complete standardization may be unattainable, necessitating flexibility to cater to diverse audience needs, priorities, and perspectives. Transparent acknowledgement of such situations is crucial.

**Systematic review registration:**

10.17605/OSF.IO/HTJMW

**Supplementary Information:**

The online version contains supplementary material available at 10.1186/s13643-026-03090-4.

## Background

The phenomenon of an evidence–policy gap is a recurring challenge, describing the discord between researchers and policy-makers in the translation of evidence into policy [1, 2]. This gap, whether due to inadequate consideration, untimeliness, or insufficient incorporation of evidence in decision-making processes, results in suboptimal resource utilization and inadequate responses to public issues [2, 3]. Conversely, policy-makers often perceive scientific advice as unrealistic, incomprehensible, or belated, driven partly by a lack of comprehension of policy processes [4]. Barriers to bridging this gap arise from inaccessible formats, political or ideological resistance to evidence, and findings that do not align with the local political context [5, 6]. Another barrier lies in the lack of understanding of the functioning of the respective spheres, in particular, the different pace between policy decision processes and the scientific knowledge generation cycle, where policy-making operates short-term, partly daily, while scientific evidence is disseminated through peer-reviewed journals [4].

Knowledge translation emerges as a discipline dedicated to addressing the evidence-policy gap [7]. Policy briefs are defined as concise documents that present research findings and recommendations to non-specialist audiences, particularly policy- and decision-makers [7, 8]. They are a well-established tool in knowledge translation alongside newer formats designed to make evidence easier to use in real-world settings. One example is the 1:3:25 model, featuring a one-page take-home message, a three-page summary, and a 25-page report [7, 8]. Strategies within knowledge translation include context-specific information, statistical data interpretation tools, and layout adaptations tailored to interestholder preferences [6, 9].

Policy briefs diverge from conventional evidence presentation. Instead, they pinpoint current problems and outline actionable research-based solutions for decision-makers [10]. By providing objective and fact-based information, policy briefs enable policy-makers to reinforce existing policies or identify areas for modification or update [11, 12]. Thus, policy briefs significantly contribute to narrowing the evidence-policy gap while acknowledging the political nature of decision-making processes [13, 14]. Key criteria for effective policy briefs encompass timing, networks, and dissemination. Timeliness ensures relevance to policy-makers when released, demanding strategic alignment with policy processes [4]. Robust networks between science and policy-makers facilitate comprehension of different professional languages and timelines, enhancing the potential impact of policy briefs [15]. Dissemination, crucial for impact [16], extends beyond distribution lists to include workshops and personal networks, requiring collaboration with dissemination partners [15].

Policy briefs originate from diverse sources, including think tanks, research organizations, universities, data infrastructures, and government agencies conducting policy-relevant research [17]. Tailored for various audiences, these briefs target policy-makers in parliament, ministerial bureaucracy, government officials, and decision influencers [16]. For instance, international organizations like the World Health Organization may produce policy briefs on global issues like the COVID-19 pandemic [18], while the Organisation for Economic Co-operation and Development may focus on health information infrastructure [17] — and both would be addressed to government officials from various Member States. Conversely, country-specific policy briefs may delve into localized concerns, like health data on obesity in Mexico [19] or country-specific policies that reflect national institutions and health system structures (such as social distancing policies during the COVID-19 pandemic in Germany) [20].

Presently, it appears that there is no standardized guidance document on how and when to develop a policy brief in health sciences [12, 13]. Click or tap here to enter text. Furthermore, those crafting policy briefs encompass a diverse spectrum, including scientists, policy-makers, communication experts, or collaborative teams of these professionals [13]. Therefore, this scoping review aims to provide an overview of recommendations and guidelines for the preparation of policy briefs. Given the diverse potential audiences, this paper concentrates on the perspective of academic health science researchers seeking to disseminate research findings to policy-makers via a policy brief.

A preliminary exploration of MEDLINE, the Cochrane Database of Systematic Reviews, and JBI Evidence Synthesis was conducted, yielding no existing or ongoing scoping reviews or systematic reviews on this subject.

## Methods

We conducted a scoping review of documents on how to write policy briefs from research to policy-makers to systematically map the evidence [21].

This scoping review followed the updated JBI guidance for scoping reviews [22]. Further, where applicable, we adhered to the Preferred Reporting Items for Systematic Reviews and Meta-analysis Protocols Extension for Scoping Reviews [PRISMA-ScR] when reporting this study [23]. Before conducting the study, a research protocol was published on the website of the Open Science Framework: https://osf.io/htjmw.

### Review questions

This review aims to answer the question: What are the recommended criteria and guidelines for structuring and presenting policy briefs generated by academic health sciences for policy-making purposes, and how do these recommendations vary across formal components and contextual considerations?

### Inclusion criteria

All documents found with the search strategy were screened according to the following inclusion criteria.

### Participants

Not applicable.

### Concept

This review focused on documents that guide the creation of policy briefs. Specifically, documents were included if they offered instructions, methods, frameworks, or advice relevant to preparing policy briefs from health sciences academia with the aim of communicating evidence to policy-makers. Documents were also included if the author’s perspective was not explicitly defined, but the guidance applied to health sciences academic researchers preparing policy briefs. The review sought to identify both general principles for policy brief development and specific practical recommendations for their preparation and dissemination.

### Context

There were no restrictions regarding the local context of the literature included.

### Types of sources

This scoping review included documents describing the preparation of policy briefs (e.g., guidance documents, handbooks, manuals, standards, guidelines, frameworks, strategies, or recommendations). Guidelines were defined as systematically developed documents containing organized recommendations intended to inform decision-making [24], informed by a structured appraisal of the best available evidence [25]. Recommendations were defined as actionable statements advising a course of action, either within guidelines or as standalone documents [24, 26]. Guidance was defined as non-binding advisory information that provides general direction, principles, or counsel to assist in understanding concepts, making decisions, or implementing practice within a specific domain [24, 27]. Abstracts, protocols, correspondence, supporting literature, and commentaries were excluded, while the authors were contacted for further information on the topic.

### Search strategy

The search strategy was designed based on the research question and follows the Peer Review of Electronic Search Strategies [PRESS] guideline [28]. We employ Boolean operators “AND” and “OR” to combine individual search steps [29], connecting main components with “AND” operators for a relevant intersection [30]. For each component, both “OR” and “AND” operators were used on suitable synonyms identified through existing study reports and literature. To narrow down the search, certain terms are searched only in the title and abstract [29]. The detailed components are delineated in Appendix I and II.

Only documents written in English, French, Spanish, or German were considered for inclusion, based solely on the authors’ linguistic proficiencies. No restrictions regarding the publication dates were placed. The literature search encompassed the PubMed, Embase, LIVIVO (SOMED), and Web of Science databases, supplemented by a manual search involving reference cross-referencing and forward citation screening. Recognizing that guidance documents might not be disseminated as traditional scientific articles, supplementary searches were conducted via Google. Contrary to our initial plans, we did not consult field experts or authors of identified literature, as we deemed the literature obtained through our search strategies sufficient. The search strategies were performed from March 01, 2023, until December 01, 2025. No publication data restrictions were applied, as preliminary searches suggested that policy briefs are a fairly recent development, with most documents emerging from approximately 2010 onward.

### Source of evidence selection

The selection of relevant articles was done in a multi-stage process. Duplicates identified were automatically removed using the reference management tool EndNote. For Google, where automation was not feasible, reviewers manually detected and removed duplicates. Subsequently, all remaining search outcomes were exported to a CSV file and integrated into a Microsoft Excel table to document the selection process. Decisions were guided by pre-specified inclusion and exclusion criteria [31]. Excluded literature was annotated with a reason for exclusion and coded numerically according to the exclusion criteria [32]. A screening of titles and abstracts was carried out to determine the potential relevance of articles in addressing the research question. Potentially relevant studies were included in the full-text screening if the title and abstract either met the inclusion criteria or did not permit a clear assessment of eligibility [33]. Finally, the full texts were assessed against the inclusion criteria. The remaining studies were included in this scoping review for data extraction and synthesis. Throughout the inclusion process, references within the included studies were examined through cross-checking and forward citation screening to determine their eligibility.

The abstract and full-text screening process step was completed independently by two researchers and compared after each step. Discrepancies were resolved through discussion until a consensus was reached or, if necessary, with the involvement of a third independent reviewer.

### Data extraction

Once all suitable publications were identified and included in this scoping review, the extraction of relevant data was initiated. Evidence was extracted using a standardized form, which was constructed based on a sample of two included publications. Two researchers pilot-tested the form. The results of the pilot testing were collectively reviewed by three researchers, and necessary changes were made to the extraction form through discussion until consensus. One researcher conducted the data extraction, which was subsequently verified by a second reviewer. Disagreements were resolved either through discussion until a consensus was reached or by involving a third reviewer.

### Data analysis and presentation

Post-data extraction, a detailed summary was formulated following the prescribed data extraction template, mapping guidelines across the two themes of (i) formal components and (ii) contextual considerations. Formal components were defined as the physical and organizational characteristics that shape how the policy brief is structured and presented to readers. Under formal components, criteria about structural and presentational aspects were delineated, including (i.i) target audience; (i.ii) length; (i.iii) layout; (i.iv) language; (i.v) point in time; and (i.vi) references. Conversely, we defined contextual components as the content, argumentation, and strategic communication approaches that convey the policy message and facilitate its uptake. It encompasses (ii.i) title; (ii.ii) summary; (ii.iii) problem statement; (ii.iv) methodology; (ii.v) results; (ii.vi) policy options; (ii.vii) recommendations; (ii.viii) implications; (ii.ix) further aspects; (ii.x) dissemination; (ii.xi) evaluation; and (ii.xii) interestholder engagement. The scoping review used a descriptive analysis methodology with frequencies calculated.

## Results

**Fig. 1:**
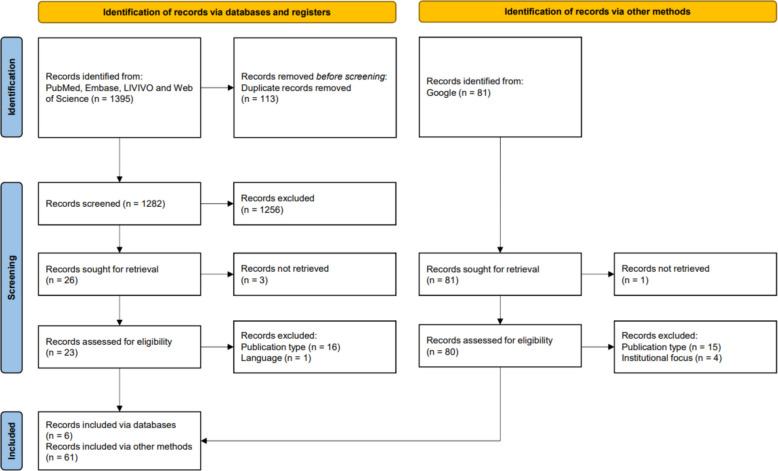
PRISMA flow chart

The search strategies yielded 1395 publications. Furthermore, we identified 81 documents through manual searches. After a full-text screening, 67 documents were deemed pertinent for inclusion, comprising 6 from scientific databases and 61 from manual searches (see Fig. 1) [11, 12, 14–16, 34–95]. The documents originated primarily from North America (24/67, 36%), Europe (22/67, 33%), and international research collaborations (13/67, 19%). Only 8 documents were included from the remaining continents (Oceania: 3/67, 4%; Asia: 3/67, 4%; Africa: 1/67, 1%; South America: 1/67, 1%). Out of the 67 included documents, 15 (22%) originated from health sciences, 16 (24%) from a related discipline, and 10 from an unrelated discipline but intended for all disciplines (13%). The largest proportion, 27 documents (40%), were either not associated with any specific discipline or did not specify a discipline (see Appendix VI).

### Formal criteria

Concerning formal criteria, 57 publications delineate the target group. Predominantly, the target group is characterized as policy-makers (35/67, 52%), supplemented by a broader category encompassing decision-makers (7/67, 10%), non-specialized groups (10/67, 15%), and various interestholder groups (5/67, 8%). The optimal length of a policy brief exhibits considerable variability, ranging from concise one-page formats to more extensive graded-entry formats spanning approximately 28 pages. Among the 52 documents specifying length recommendations (77%), nearly half (25/67, 37%) advocate a succinct range of 2 to 8 pages. Approximately 20% of the documents (14/67) address the temporal dimension of policy briefs, with 9 publications (13%) advocating a sense of urgency or alignment with pivotal moments such as imminent decisions or crises. Two documents assert the temporal variability of policy briefs contingent upon the specific aims of the discourse.

Recommendations concerning layout (55/67, 82%) show homogeneity, endorsing the incorporation of marketing mechanisms or/-branding through highlights, a color scheme, data visualization (45/67, 67%), and overall appeal to the target audience (8/67, 12%). Similarly, consensus prevails in linguistic considerations (58/67, 88%), wherein simplicity and clarity are underscored (34/67, 51%), eschewing technical jargon (34/67, 51%), and tailoring the language to the target group (24/67, 36%). A unified approach to language use in policy briefs, favoring active language (5/67, 7%), minimal acronyms (5/67, 7%), or explicating them when employed (5/67, 7%), is apparent. Regarding referencing, a majority of publications (43/67, 64%) categorically stipulate their mandatory inclusion. However, disparities arise concerning the requisite volume of references, ranging from a stipulated maximum of between 1 and 7 references (11/67, 16%) to the inclusion of all references used in the research (12/67, 18%). Furthermore, a subset of documents (18/67, 27%) recommends supplementary literature for further reading. The distribution of formal criteria within the 67 included publications is shown in Table 1.

**Table 1:** Overview of formal criteria by frequency in absolute numbers and percent (*N* = 67), with categories in bold and subcategories in italic

*Category*	*N*	*%*
**Target audiences**	**57**	**86**
* Policy-maker*	35	53
* Decision-maker*	7	10
* Non-specialized groups*	10	15
* Further interestholder groups*	5	8
**Length**	**52**	**78**
* Less than 2 pages*	5	7
* 2–8 pages*	25	37
* More than 8 pages*	8	11
**Point in time**	**14**	**21**
* Urgency/momentum*	9	13
* Depending on the aim*	2	3
**Layout**	**55**	**82**
* Branding, though highlights, color scheme, visualizations*	45	67
* Appealing for target group*	8	12
**Language**	**58**	**87**
* Simple/clear*	34	51
* No technical jargon*	34	51
* Target group specific*	24	36
* Active voice*	5	7
* No to little acronyms*	5	7
* Write out acronyms*	5	7
**References**	**43**	**64**
* Maximum of 1 to 7 references*	11	16
* Inclusion of all references used*	12	18
* Inclusion of additional references for further reading*	18	27

### Contextual considerations

At the outset, according to 55 documents (82%), it is recommended that policy briefs articulate a concise (40%), descriptive (36%), and captivating (36%) title, thereby enticing readers to engage further with the content. In terms of further contextual considerations, 49 documents (75%) advocate commencing with a summary, manifested either as an executive summary (28/67, 41%), key messages (14/67, 21%), or both (10/67, 15%).

### Background, methodology, and research

The subsequent section may include a problem statement (61/67, 91%). This may encompass an introduction and context (37/67, 55%), followed by an explication of the key topic and its description (50/67, 75%). The section may also cover causes of the chosen topic/phenomenon (21/67, 31%), its consequences (18/67, 27%), and significance (36/67, 54%). A smaller subset of documents incorporates the declaration of the policy brief’s aim (11/67, 16%), methodology (3/67, 4%), limitations (1/67, 1%), and results and recommendations (6/67, 9%) within the problem outline. Methodological considerations as a section are stated in 26 documents (39%), with 14 prescribing its inclusion as mandatory (21%), 4 deeming it optional (6%), and one document advising against its inclusion (1%). The methodology may be appended as an appendix (2/67, 3%) or integrated into another segment (9/67, 13%). A subsequent section, as evidenced by 25 documents (37%), pertains to the inclusion of research results, with 24 characterizing them as “classical results” (36%), adhering to established standards for academic publication, and incorporating implications (6/67, 9%). Additional facets such as the problem’s impact (3/67, 4%), an overview of the academic landscape (2/67, 3%), a critique of prevailing political solutions, and options for addressing the core problem (5/67, 7%) may also be explicated. Some documents (11/67, 16%) recommend using statistics to demonstrate the problem’s magnitude and provide evidence. The statistical information ought to be presented in easily comprehensible formats, such as visual depictions, and should be confined to pertinent essentials. 40 documents (40/67, 60%) described policy options, tackling the issue at stake as the next step, in which one (3/67, 4%) or several (32/67, 48%) policy options are described and weighed up based on criteria (13/67, 19%).

### Recommendations for policy-making

Subsequently, the analysis may encompass the categorization of recommendations (57/67, 85%) into singular instances (6/67, 9%), multiple recommendations (17/67, 25%), or a minimum of one (3/67, 4%). These recommendations may adopt a procedural framework, delineating the specific responsibilities assigned to interestholders within the policy option (21/67, 32%). In 15 documents (22%), the delineation of implications is advocated, varying in its intent. Implications may manifest as an implementation guide (6/67, 9%), articulating the consequences of the proposed recommendation (4/67, 6%), or serving as a surrogate for recommendations (3/67, 4%), particularly in cases where the target audience did not ask for recommendations. Approximately half of the documents (36/67, 54%) suggest the incorporation of supplementary content, encompassing contact information (21/67, 31%), acknowledgements and disclaimers (13/67, 19%), an appendix (11/67, 16%), a hyperlink to the comprehensive study or the organization’s website (9/67, 13%), and the inclusion of the policy brief’s objective as an autonomous section (5/67, 7%).

### Dissemination, evaluation, and stakeholder engagement

The process of developing a policy brief may extend beyond the act of composition. Interestholder engagement emerges as a strategy (12/67, 18%) encompassing activities such as review (2/67, 3%), option prioritization (1/67, 1%), policy brief dissemination (4/67, 6%), and the augmentation of impact (1/67, 2%). Dissemination strategies (22/67, 33%) may involve the circulation of the policy brief to the target audience (14/67, 21%), social media publications (11/67, 16%), or alternative platforms (6/67, 9%) such as the organization’s website or journals. Additional methods may include presenting the policy brief at events (10/67, 15%), implementing follow-up mechanisms (3/67, 4%), or adopting a comprehensive approach combining multiple methodologies for dissemination (16/67, 24%). Four documents advocate for the incorporation of evaluation strategies to assess impact, employing methodologies like surveys (1/67, 1%), quantitative indicators (1/67, 1%), or comprehensive short- and long-term evaluations using both qualitative and quantitative metrics (1/67, 1%).

## Discussion

We provide an overview of documents describing the structure, format, length, layout, and key characteristics of policy briefs to inform policy decision-making from health science academia. Across the 67 included documents, we observed a high level of variability in structure, content, and methodological detail. While many documents share core recommendations (such as the use of clear and non-technical language, visual branding, and inclusion of concise summaries, issue identification, and recommendations), there is considerable variation in content, including how to present the key issue, how many recommendations to include, and which additional components must be provided. Additionally, several areas only received limited attention, such as timing, dissemination strategies, evaluation methods, and interestholder engagement (see Tables 1 and 2). Lastly, the included documents were highly varied in factors such as length and detail, which led to a comparison of one one-pager against longer publications (see Appendix V).

**Table 2:** Overview of contextual considerations by frequency in absolute numbers and percent (*N* = 67), with categories in bold and subcategories in italic

*Category*	*N*	*%*
**Title**	**55**	**82**
**Summary**	**50**	**75**
* Executive summary*	28	41
* Key messages*	14	21
* Both*	10	15
**Problem statement**	**61**	**91**
* Introduction/Context*	37	55
* Topic description*	50	75
* Causes of the issue*	21	31
* Consequences of the issue*	18	27
* Significance*	36	54
* Aim of the policy brief*	11	16
* Methodology*	3	4
* Limitations*	1	1
* Results and recommendations*	6	9
**Methodology**	**26**	**39**
* Core component*	14	21
* Optional*	4	6
* Not to be included*	1	1
* Included in the Appendix*	2	3
* Integrated in other sections*	9	13
**Results**	**25**	**37**
* Impact of the issue*	3	4
* Research results (“academic way of presenting findings”)*	24	36
* Implication of results*	6	9
* Critique of current political solutions*	2	3
* Results as a replacement for policy options*	5	7
**Policy options**	**40**	**60**
* Optional component*	1	1
* Presenting one policy option*	3	4
* Presenting multiple policy options*	32	48
* Comparing/Weighing up options based on criteria*	13	20
**Recommendations**	**57**	**85**
* One recommendation*	6	9
* Multiple recommendations*	17	25
* Both possible*	3	4
* Step-by-step guide for implementing recommendations*	21	32
**Further components**	**36**	**54**
* Contact information*	21	31
* Acknowledgements and disclaimer*	13	19
* Appendix*	11	16
* Hyperlink to full-text*	9	13
* Aim of the policy brief*	5	7
**Dissemination**	**22**	**33**
* Sending/Handing-out*	14	21
* Social media*	11	16
* Events*	10	15
* Publish*	6	9
* Multi-method strategy*	16	24
* Follow-up*	3	4
**Evaluation**	**4**	**6**
* Necessary*	4	6
* Quantitative indicators*	1	1
* Qualitative and qualitative indicators*	1	1
* Survey*	1	1
**Interestholder engagement**	**12**	**18**
* Reviewing policy brief*	2	3
* Assisting in prioritizing options*	1	1
* Dissemination*	4	6
* Strengthening impact*	1	1

### Variability in reporting requirements

The observed variability may be traced to several interconnected factors. One primary contributor to this variability may be the divergent conceptualization of what a “policy brief” is and what purpose it serves [15, 52]. Across the analyzed documents, conceptualizations ranged along a spectrum: from more evidence-focused synthesis to advocacy-oriented documents designed to influence policy choices [13, 16]. This definitional spread reflects both the institutional mandates and disciplinary traditions of authors.

Another driver of variation concerns the intended audience. Included publications often broadly reference “decision-makers”, yet the term is ill-defined and likely depends on the context [13]. The audience might differ in expertise, available time, and tolerance for technical content [13, 96]. This may partially explain why some sets of guidance or guidelines emphasize brevity and a concise format, while others prioritize explanatory context and detailed analysis, resulting in divergent formulations of “best practice” [10, 13, 16, 64]. Importantly, few documents clearly specify their intended audience [35, 36, 51, 52, 62, 69, 74, 80, 81, 97], making it difficult to judge whether variation reflects purposeful adaptation or inconsistent advice [98, 99].

While our focus was primarily on documents originating from the health sciences academia, only 31 documents emerged from health sciences or a related discipline. This is because we also included those aimed at researchers across various disciplines, despite the authoring organizations originating from diverse fields such as sociology or environmental sciences, as well as those that did not specify a disciplinary background (see Appendix VI). Different disciplines might hold different assumptions about what constitutes credible or policy-relevant evidence [100, 101]. These epistemic differences might have influenced how evidence is summarized and justified in policy briefs [100], although such assumptions were not made explicit in the documents reviewed.

Geographical variations may also have contributed to the variability observed. Our review included documents from all continents, which may be advantageous because successful knowledge translation relies on tailoring to local contexts to enhance relevance and uptake [102, 103]. However, as no document reported on specific geographical adaptations, combined with an uneven regional representation (see Appendix VI), makes it difficult to determine the extent to which the observed variation reflects purposeful contextualization. Nevertheless, the variation observed may indicate increasing attention to local priorities in the development of documents for evidence communication [104].

The temporal dimension of policy-making introduces another layer of complexity. The dynamic nature of policy-making and the evolving understanding of effective knowledge translation communication necessitate continuous updates [4, 105, 106]. Therefore, the understanding of effective evidence communication has evolved, moving toward conciseness, visual summaries, and narrative storytelling [107, 108]. Consequently, older guidelines may differ due to changes in both political and media communication norms. While some early frameworks, such as Lavis et al. (2009) [64] already proposed short 6–8-page formats with executive summaries, more recent models incorporate infographics, plain language summaries, and digital dissemination elements [91, 92].

Additionally, the distinction between policy briefs based on specific research projects and those summarizing broader thematic evidence might further explain variation. The former tends to emphasize methods and results, while the latter typically foregrounds context and interpretative synthesis [13, 109]. Although the included documents rarely distinguished between these origins, potential differences in emphasis, scope, and contextual relevance may contribute to variability in the recommended approaches [110].

### Implications of variability

The identified variability within policy brief preparation documents presents challenges for researchers who seek unambiguous and consistent direction in crafting impactful policy briefs. The definitional differences, following different instructions, may yield different outputs — each labelled as policy briefs, but not necessarily aligned in form, purpose, or audience [12]. This inconsistency might leave researchers uncertain about which format is most appropriate for their goals, undermine comparability, and risk that some policy briefs are overlooked or dismissed by policy-makers because they do not align with expected formats [13, 111].

The central paradox emerging from these findings is the tension between standardization and flexibility [12–14, 112, 113]. Standardization can enhance legitimacy, facilitate training, and strengthen recognition of policy briefs as professional tools [114, 115], while providing a shared framework grounded in research on knowledge translation and evidence communication [115]. Such frameworks may help ensure that policy briefs consistently present information in ways that align with the audience’s needs [116, 117]. Yet, excessive uniformity might risk producing overly generic documents that lack audience sensitivity, contextualization, and nuance required for political relevance [64, 112, 117]. Flexibility, by contrast, can allow adaptation to complex and shifting policy environments, etc. [10, 64, 118], but unchecked flexibility may fragment identity and limit opportunities for mutual learning across institutions [13, 112]. Within academic institutions, evidence communication is often organized at the level of individual departments, research groups, or projects rather than through a coordinated institutional approach [119]. While this enables tailoring to specific audiences, it might also lead to fragmentation, as teams independently develop formats and styles, resulting in missed opportunities for collective learning and the systematic development of shared standards [119, 120].

Lastly, it is important to acknowledge the existing gap in the evaluation of most documents and the broad spectrum of thematic areas to which policy briefs are applied. Very few guidelines show evidence of systematic testing (see Table 2), and it remains unclear whether the evaluations and learnings available apply to other documents and contexts. The absence of a standardized mechanism to assess the impact and effectiveness of different approaches may further complicate efforts to discern improvements and support systematic refinement over time [13].

Overall, variability appears to be an inherent feature of evidence communication, but one that requires further management. For academic authors’ engaging with health policy, the challenge appears to be to retain academic accountability while adjusting style and structure to policy realities [10, 13, 121]. Understanding variability as an intrinsic and context-driven feature can shift the focus from enforcement of uniform rules toward the design of adaptable principles that support coherence, credibility, and responsiveness simultaneously [121].

### Potential strategies

Addressing the observed, partly unknown variability may assist in achieving higher levels of impact and fostering a more uniform understanding of policy briefs [122]. Rather than treating variability primarily as a deficit, insights from knowledge translation and implementation science suggest that variability can be addressed through structured processes that balance shared standards with contextual adaptation [13, 123]. Nonetheless, it is important to acknowledge the absence of methodological sections within the included documents. Therefore, it remains unclear whether they have already used collaborative approaches, and if so, to what extent.

A promising pathway is the adoption of an advice co-development approach, whereby all relevant actors, such as researchers, policy-makers, policy intermediaries, and other intended end-users, are systematically involved in developing documents on how to write policy briefs [54, 123, 124]. This approach aligns with integrated knowledge translation principles, which position knowledge users as equal partners across the research process rather than passive recipients at the endpoint [13, 123, 125, 126]. Previous literature indicates that early and sustained engagement of policy-makers enhances the relevance, usability, and legitimacy of evidence products by aligning them more closely with the audience’s needs, constraints, and interpretive frames [2, 113, 117, 126].

Co-development processes can be operationalized through established participatory techniques designed to integrate diverse perspectives and reach agreement in complex contexts such as guideline development [54, 127, 128]. Methods such as consensus conferences, Delphi methods, and facilitated collaboration workshops are widely used to integrate diverse forms of expertise and build shared understanding across heterogeneous interestholder groups [127, 129, 130]. Applied to documents on how to write a policy brief, these techniques may support collective deliberation on core principles, intended functions, and acceptable degrees of flexibility, while ensuring that guides reflect both scientific standards and policy realities. Comparable approaches have been proposed previously as mechanisms to enhance the usability and uptake of evidence products, including policy briefs [13, 54, 126].

Therefore, this approach might not only be useful to policy briefs as a tool but also be embedded within a combination of strategies such as dialogues, stakeholder consultations, technical reports, and advisory committees, etc. [10, 64, 115]. This perspective may be particularly relevant in settings where policy briefs are less routinely produced or used, including low- and middle-income countries, fragile governance contexts, or highly decentralized policy systems, where informal relationships, intermediaries, and political timing may exert greater influence than written products alone [92, 127].

Within such participatory frameworks, a key objective may include the definition of minimum shared standards that articulate essential qualities of credible policy briefs without prescribing rigid templates [121, 131]. Similar to the homogeneity found within this scoping review, shared standards could include factors such as concise length, key messages, policy recommendations, and a visually identifiable design (see Tables 1 and 2). Rather than enforcing uniform formats, consensus-based standards can delineate which elements are considered core and which may be adapted to the context, audience, or disciplinary norms [132]. This “core-and-flexible” approach is consistent with implementation science principles of fidelity and adaptation, which emphasize preserving essential functions while allowing form to vary in response to contextual conditions [117, 131]. By explicitly addressing adaptation, guidance can support authors in tailoring briefs to specific policy timelines, governance levels, or stakeholder audiences [16], and without undermining overall coherence and impact.

Beyond structure and content, co-development provides an opportunity to strengthen attention to processes that remain underrepresented in existing documents, particularly dissemination and evaluation (see Appendix VI). Although dissemination strategies were mentioned in only about one-third of the included documents, they may influence whether policy briefs reach relevant audiences and enter decision-making arenas [13, 113]. Similarly, while direct attribution between a policy brief and policy change is rarely feasible [128], evaluation might serve as a learning mechanism to improve clarity, usability, and dissemination [34, 128]. However, it must account for the realities of governance where outcomes depend on political timing, networks, and competing agendas [3]. Formative evaluation, feedback loops, and pilot testing may yield practical insights without burdening authors with unrealistic expectations of measuring policy impact [3, 126]. In this sense, evaluation does not quantify influence but might support iterative refinement and collective learning, reflecting the knowledge translation principle that improvement depends on dialogue, not on metrics alone [5, 10, 34].

Greater emphasis on co-development and engagement may raise normative questions about the role of academics in policy-making [133]. Influencing policy through evidence advocacy necessitates navigating various networks and identifying windows of opportunity, potentially blurring the line between scientists and policy-makers [3, 133]. Ongoing debates within the literature highlight the need to balance responsiveness to policy needs with adherence to scientific standards [13, 120, 134]. Within co-development, this tension may be addressed by prioritising responsiveness to the end-user’s needs without adopting a prescriptive stance on how evidence should be used, thereby maintaining scientific integrity while enhancing relevance [118].

Finally, certain included documents appeared to be particularly useful for writing robust policy briefs. Despite the ongoing limitations for future researchers, we identified three documents that might serve as a good starting point for writing a policy brief. In our opinion, the publications authored by the Mental Health Innovation Network [68], Lavis, Boyko et al. [64] and Antonopoulou et al. [34] appeared to be the most useful. These frameworks provided the most detailed guidance on both structural elements and practical implementation considerations, making them particularly valuable to researchers new to policy brief development who require comprehensive step-by-step guidance.

### Limitations

By utilizing the non-scientific search engine Google, which yields billions of search results, it was impractical to screen every result comprehensively. The screening was conducted until no (potential) hits were identified across multiple pages. Nevertheless, it cannot be ruled out that additional eligible documents may exist on Google, which may have limited the reproducibility of this research, as search algorithms are dynamic and results may vary over time and by location. However, given that most included documents (61/67) were identified through this search engine, their inclusion remained essential to achieving coverage of available documents, even though they were not peer reviewed.

Although we applied no restrictions regarding the geographic context of the studies, the findings may still have been constrained by language. Despite drawing on all language competencies available within the research team, it is likely that we inadvertently excluded publications from countries in which none of the languages included in our search are widely spoken as native languages.

Due to the high volume of hits, data extraction was performed by a single reviewer, with an independent verification conducted by a second reviewer. While this approach aligns with recommendations for large-scale scoping reviews and the updated JBI guidance on scoping reviews, it may have increased the risk of errors during the extraction process [135].

Inclusion criteria, specifically the imperative of neutrality concerning both author and end-user, led to the incorporation of documents originating from non-health science institutions. This inclusion was imperative in light of the pre-defined inclusion criteria and the published study protocol. Notably, very few documents were solely designed for academic health sciences. Therefore, this may have contributed to the variability observed.

## Conclusion

This scoping review aimed to provide an overview of documents on how to write a policy brief. While finding a range of homogeneous criteria, such as clear language, visual branding, and providing recommendations, we also identified substantial variability across existing documents. While diversity in approaches can be valuable, the degree of variability identified may challenge the usefulness and acceptance of policy briefs: Decision-makers may struggle to navigate inconsistent formats, and researchers may face uncertainty about essential components. These inconsistencies may hinder collaboration, uptake of evidence, and ultimately weaken the impact of policy briefs.

At the same time, our findings point to the tension between both standardization and flexibility. It may be beneficial to anchor the documents in shared standards while remaining adaptable to different contexts, timelines, types of evidence, and target audiences. To support this, we recommend a co-development approach that involves relevant interestholders in defining core standards alongside flexible components. Such processes may also explore evaluation mechanisms, such as formative feedback or iterative refinement, to ensure continued relevance and improvement. Lastly, greater transparency in how the documents were developed might strengthen credibility and usability, as most included documents did not report their methodology.

## Supplementary Information

Supplementary Material 1.

## Data Availability

Not applicable.
