# Supplementary material for: Guidelines and recommendations for preparing policy briefs from research into policy-making in health sciences: a scoping review
Source: Syst Rev. 2026 Feb 9;15:80. doi: 10.1186/s13643-026-03090-4 (PMC12983617; doi:10.1186/s13643-026-03090-4)
Supplement: Supplementary file 1 — Supplementary Material 1. [file 13643_2026_3090_MOESM1_ESM.docx]

# **Appendix**

Appendix I: Search Strategy for PubMed

(“Policy brief” [Title/Abstract] OR “Policy briefs” [Title/Abstract] OR “evidence brief”[Title/Abstract] OR “evidence briefs”[Title/Abstract] OR “issue brief”[Title/Abstract] OR “issue briefs”[Title/Abstract] OR “policy note”[Title/Abstract] OR “policy notes”[Title/Abstract] OR “policy analysis brief”[Title/Abstract] OR “policy analysis briefs”[Title/Abstract] OR “research brief”[Title/Abstract] OR “research briefs”[Title/Abstract])

AND

(Guidance [Title/Abstract] OR Guideline [MeSH] OR Manual [Title/Abstract] OR Manual [MeSH] OR Guide* [Title/Abstract] OR Handbook [Title/Abstract] OR Instruct* [Title/Abstract] OR recommend*[Title/Abstract] OR consen*[Title/Abstract] OR statement*[Title/Abstract] OR framework*[Title/Abstract] OR strategy[Title/Abstract] OR strategies[Title/Abstract] OR standard*[Title/Abstract])

Appendix II: Search Strategy for EMBASE

((‘Policy brief’:ti,ab,kw OR ‘Policy briefs’:ti,ab,kw OR ‘evidence brief‘:ti,ab,kw OR ‘evidence briefs‘:ti,ab,kw OR ‘issue brief‘:ti,ab,kw OR ‘issue briefs‘:ti,ab,kw OR ‘policy note‘:ti,ab,kw OR ‘policy notes‘:ti,ab,kw OR ‘policy analysis brief‘:ti,ab,kw OR ‘policy analysis briefs‘:ti,ab,kw OR ‘research brief‘:ti,ab,kw OR ‘research briefs‘:ti,ab,kw)

AND

(Guidance:ti,ab,kw OR Guideline:ti,ab,kw OR Manual:ti,ab,kw OR Guide:ti,ab,kw OR Handbook:ti,ab,kw OR Instruction:ti,ab,kw))

Appendix III: Search Strategy for LIVIVO

(TI,AB,KW:("policy brief" OR "policy briefs" OR

"evidence brief" OR "evidence briefs" OR

"issue brief" OR "issue briefs" OR

"policy note" OR "policy notes" OR

"policy analysis brief" OR "policy analysis briefs" OR

"research brief" OR "research briefs"))

AND

(TI,AB,KW:(guidance OR guideline OR manual OR guide OR handbook OR instruction))

Appendix IV: Search Strategy for Web of Science

TS=("policy brief" OR "policy briefs" OR

"evidence brief" OR "evidence briefs" OR

"issue brief" OR "issue briefs" OR

"policy note" OR "policy notes" OR

"policy analysis brief" OR "policy analysis briefs" OR

"research brief" OR "research briefs")

AND

TS=(guidance OR guideline OR manual OR guide OR handbook OR instruction)

Appendix V: Search Strategy for Google

((‘Policy brief OR ‘Policy briefs’))

AND

(Guidance: OR Guideline OR Manual OR Guide OR Handbook OR Instruction))

Appendix VI: Depiction of included publications and reported items

| Scientific publication | Grey literature |  | Target audience | Length | Point in time | Layout | Language | References | Title | Key messages | Executive summary | Problem description | Methodology | Results | Policy options | Recommendation | Implication | Additional information | Dissemination | Evaluation | Stakeholder Engagement | Geography | Discipline |
| --- | --- | --- | --- | --- | --- | --- | --- | --- | --- | --- | --- | --- | --- | --- | --- | --- | --- | --- | --- | --- | --- | --- | --- |
|  | X | Antonopoulou et al. (34) | X | X | X | X | X | X | X | X | NA | X | (X) | X | NA | X | X | X | X | X | X | Int | NA |
|  | X | Australian National University (35) | NA | X | NA | X | X | X | X | X | X | X | NA | X | NA | X | NA | NA | NA | NA | NA | Oc | NA |
|  | X | Baragan (36) | NA | X | NA | X | X | X | X | X | X | X | (X) | (X) | X | X | NA | X | NA | NA | NA | Int | + |
| X |  | Benton et al. (37) | X | X | X | X | X | X | X | NA | NA | X | NA | NA | X | X | NA | X | NA | NA | NA | Int | + |
|  | X | Biodiversa+ (38) | X | X | X | X | X | X | NA | X | NA | NA | NA | NA | NA | X | NA | X | X | NA | X | Eur | o |
|  | X | Boston University – School of Public Health (39) | X | NA | NA | X | NA | NA | X | NA | NA | X | NA | NA | X | X | NA | NA | NA | NA | NA | No | ++ |
|  | X | Centre College (43) | X | NA | NA | NA | NA | X | X | (X) | X | X | NA | NA | X | X | NA | X | NA | NA | NA | No | NA |
|  | X | Centre for Arctic Policy Studies (41) | X | X | NA | NA | NA | X | X | X | NA | X | X | NA | X | NA | NA | NA | NA | NA | NA | No | o |
|  | X | Centers for Disease Control and Prevention (41) | X | NA | NA | X | X | NA | NA | NA | NA | NA | NA | NA | NA | NA | NA | NA | NA | NA | NA | No | ++ |
|  | X | Center for Health Economics and Policy (40) | NA | X | NA | X | X | X | X | X | NA | X | X | X | X | X | NA | X | NA | NA | NA | No | ++ |
|  | X | Chr. Michelsens Institut (93) | X | X | NA | X | X | X | X | X | NA | X | X | X | NA | X | (X) | NA | X | NA | NA | Eur | NA |
|  | X | Community-Based Monitoring System (CBMS) Network Coordinating Team (48) | X | X | NA | X | X | X | X | NA | X | X | NA | NA | X | X | NA | (X) | NA | NA | NA | As | o |
|  | X | Cooper & McCoy Roth (50) | X | X | NA | X | X | X | X | X | X | X | (X) | X | X | NA | X | X | NA | NA | NA | No | + |
|  | X | Culture Partnership (51) | NA | X | NA | NA | NA | X | NA | (X) | X | X | NA | NA | X | X | NA | X | X | NA | NA | Eur | NA |
|  | X | Dagenais & Ridde (52) | NA | X | NA | X | NA | X | X | X | X | X | NA | X | NA | X | NA | NA | NA | NA | NA | Int | NA |
| X |  | DeMarco & Adams Tufts (53) | X | X | X | X | X | X | X | NA | X | X | NA | X | X | X | NA | NA | NA | NA | NA | No | + |
| X |  | Dunlop et al. (54) | X | NA | NA | NA | NA | X | X | X | NA | X | NA | X | NA | X | X | X | NA | NA | NA | Int | NA |
| X |  | Ffrench-Constant (55) | X | X | X | X | X | X | NA | NA | X | X | X | X | NA | X | NA | X | X | NA | NA | Eur | NA |
|  | X | Food Security Committee (56) | X | X | NA | X | X | X | X | X | (X) | X | NA | NA | X | X | (X) | X | X | NA | NA | Int | + |
|  | X | Gal (57) | X | X | NA | X | X | NA | X | NA | X | X | (X) | X | NA | X | (X) | NA | X | NA | NA | Afr | NA |
|  | X | Habib University (80) | NA | NA | NA | NA | NA | X | NA | NA | X | X | X | X | NA | X | NA | NA | NA | NA | NA | As | NA |
|  | X | Harvard University (58) | X | X | NA | X | X | X | NA | NA | NA | X | (X) | NA | X | X | NA | NA | NA | NA | NA | No | ++ |
|  | X | International Centre for Policy Advocacy (45) | X | X | NA | X | X | X | X | NA | X | X | NA | NA | X | X | NA | X | NA | NA | NA | Eur | o |
|  | X | International Development Research Centre, Centre de recherches pour le développement international (44) | X | X | NA | X | X | NA | X | NA | X | X | (X) | (X) | NA | X | (X) | NA | NA | NA | NA | No | + |
|  | X | Jarman (59) | X | NA | NA | X | X | NA | NA | X | NA | NA | NA | NA | NA | NA | NA | NA | NA | NA | NA | No | ++ |
|  | X | Jones and Walsh (14) | X | NA | NA | X | X | X | NA | NA | NA | NA | NA | NA | NA | X | X | NA | X | NA | NA | Eur | + |
|  | X | JPI Urban Europe (60) | X | X | X | X | X | X | X | X | X | X | NA | (X) | X | X | X | X | X | NA | NA | Eur | + |
| X |  | Keepnews (61) | X | X | NA | NA | NA | X | X | NA | X | X | NA | NA | X | X | NA | NA | NA | NA | NA | No | NA |
|  | X | Kheirandish (62) | NA | X | NA | X | X | X | X | X | (X) | X | X | NA | X | X | NA | X | NA | NA | NA | Int | ++ |
|  | X | Kobzar (63) | X | X | NA | X | X | X | X | (X) | X | X | (X) | X | NA | X | (X) | (X) | NA | NA | NA | Eur | NA |
| X |  | Lavis, Boyko, et al. (64) | X | X | NA | NA | X | X | X | X | NA | X | (X) | NA | X | NA | NA | X | NA | X | X | Int | ++ |
|  | X | Leipniz ZMT (65) | X | X | X | X | X | X | X | NA | X | X | NA | X | (X) | X | NA | NA | X | NA | X | Eur | o |
|  | X | Lund (66) | X | X | NA | NA | NA | X | NA | NA | NA | X | X | X | NA | X | (X) | NA | NA | NA | NA | Eur | NA |
|  | X | McIvor (67) | X | X | NA | X | X | NA | X | (X) | (X) | X | NA | NA | X | X | NA | NA | NA | NA | NA | No | NA |
|  | X | Model Systems Knowledge Translation Center (MSKTC) (69) | NA | NA | NA | NA | X | NA | X | NA | NA | X | NA | X | NA | X | (X) | X | NA | NA | NA | No | ++ |
|  | X | Mental Health Innovation Network (68) | X | NA | X | X | X | X | X | X | X | X | (X) | X | NA | (X) | NA | X | X | NA | X | Eur | ++ |
|  | X | Netherlands Organisation for Scientific Research (94) | X | X | NA | X | X | NA | X | X | X | X | (X) | X | (X) | X | (X) | NA | NA | NA | NA | Eur | NA |
|  | X | Neumann & Reed (70) | X | X | NA | X | X | X | X | X | X | X | NA | X | NA | X | NA | X | X | NA | X | Int | NA |
|  | X | Northern Bridge (71) | X | X | NA | X | X | NA | X | X | X | X | NA | NA | X | X | (X) | X | X | NA | NA | Int | + |
|  | X | Overseas Development Institute (72) | X | (X) | NA | X | X | X | X | (X) | X | X | (X) | X | NA | X | (X) | NA | NA | NA | NA | Eur | + |
|  | X | Pacific Community – Public Health Division (49) | X | X | NA | X | X | X | X | NA | X | X | NA | X | X | X | X | NA | NA | NA | NA | As | ++ |
|  | X | Parkers (73) | X | X | NA | X | X | NA | X | NA | NA | X | NA | NA | X | (X) | NA | NA | X | NA | NA | Eur | o |
|  | X | Posner (74) | NA | NA | NA | X | X | NA | NA | NA | NA | X | NA | NA | X | NA | NA | (X) | X | NA | NA | No | o |
|  | X | Queen Mary University of London – Centre for Public Engagement (47) | X | X | X | X | X | X | X | X | X | X | X | NA | NA | NA | X | X | X | NA | X | Eur | o |
|  | X | Quorum Analytics (75) | X | X | X | X | X | X | X | X | X | X | NA | X | X | X | NA | X | NA | NA | NA | No | NA |
|  | X | Rural Health Research Gateway (76) | X | X | NA | X | X | X | X | X | (X) | X | X | X | NA | X | (X) | NA | NA | NA | NA | No | ++ |
|  | X | Sociology Policy Briefs (95) | X | X | NA | NA | X | X | X | NA | X | X | X | X | X | X | X | X | NA | NA | NA | Int | + |
|  | X | Stowe (77) | X | NA | NA | X | X | X | X | (X) | X | X | X | NA | X | X | NA | X | NA | NA | NA | No | NA |
|  | X | Tabbara (78) | X | X | NA | X | X | NA | NA | NA | NA | X | Y | NA | X | X | NA | NA | NA | NA | NA | Int | o |
|  | X | The Australian Prevention Partnership Centre (46) | X | X | NA | X | X | X | X | X | X | X | X | X | NA | X | (X) | X | NA | NA | NA | Oc | ++ |
|  | X | The SURE Collaboration (79) | X | X | X | NA | NA | X | X | X | X | X | X | NA | X | NA | NA | (X) | X | X | X | Int | + |
|  | X | Trent University (81) | NA | NA | NA | X | X | X | X | NA | X | X | NA | NA | X | X | NA | NA | NA | NA | NA | No | NA |
|  | X | UK Parliament (83) | X | NA | NA | X | X | X | X | X | NA | NA | NA | NA | NA | NA | NA | X | NA | NA | X | Eur | NA |
|  | X | University College Davis (86) | X | X | NA | X | X | NA | NA | X | (X) | X | NA | X | X | X | X | NA | NA | NA | NA | No | NA |
|  | X | University of Exeter (87) | X | X | NA | X | X | NA | NA | NA | NA | X | NA | X | NA | X | X | X | NA | NA | NA | Eur | NA |
|  | X | University of Iowa (15) | X | X | X | X | X | X | X | (X) | (X) | X | (X) | X | X | X | NA | X | X | X | X | No | NA |
|  | X | University of North Carolina at Chapel Hill (88) | X | NA | NA | X | X | X | X | (X) | X | X | NA | NA | X | X | NA | X | NA | NA | NA | No | NA |
|  | X | University of Reading (89) | X | X | NA | X | X | X | X | (X) | X | X | NA | X | NA | X | NA | X | X | NA | X | Eur | NA |
|  | X | University of Waterloo (90) | X | X | NA | X | X | X | NA | (X) | X | NA | NA | NA | X | X | NA | NA | NA | NA | NA | No | NA |
|  | X | University of Wollogong (91) | X | NA | NA | X | X | X | X | NA | X | X | NA | X | X | X | NA | NA | NA | NA | NA | Oc | NA |
|  | X | Weyrauch & D‘Agostino (92) | X | X | X | X | X | NA | X | (X) | X | X | NA | NA | X | X | X | NA | NA | NA | NA | Sa | ++ |
|  | X | Wolfe (93) | X | X | X | X | X | X | X | X | X | X | (X) | X | NA | X | (X) | X | X | NA | X | Eur | ++ |
| X |  | Wong (12) | X | X | NA | X | X | NA | X | X | NA | X | NA | NA | X | NA | NA | NA | NA | NA | NA | No | ++ |
|  | X | Yeakey (94) | X | X | NA | X | X | X | NA | X | X | X | NA | X | NA | X | (X) | NA | NA | NA | NA | No | + |
|  | X | Young and Quinn (16) | X | X | NA | X | X | X | X | NA | X | X | (X) | X | X | X | NA | X | X | NA | NA | Eur | + |
|  | X | Young, and Quinn (95) | X | X | NA | X | X | X | X | (X) | X | X | NA | NA | X | X | NA | (X) | NA | NA | NA | Eur | + |
|  | X | Young & Quinn (96) | X | X | NA | X | X | X | X | NA | X | NA | X | X | X | X | NA | X | X | NA | NA | Eur | + |

'X' denotes pertinent extracted contents, with the notation '(X)' indicating that said contents were either assimilated into other sections or held as optional for inclusion within the policy brief. 'Y' signifies contents that were recommended against, and 'NA' denotes instances where the publication did not contain the specified item. For “Geography”, ‘int’ refers to documents written in international collaborations, ‘Eur’ to Europe, ‘No’ to North America, ‘Sa’ to South America, ‘Af’ to Africa, and ‘Oc’ to Oceania. In “Discipline” ‘++’ denotes an authoring institution/research team from health sciences, ‘+’ a discipline related, and ‘o’ authoring organization from an unrelated discipline with a document applicable to all disciplines.
